# A New Mouse Model for the Study of Human Breast Cancer Metastasis

**DOI:** 10.1371/journal.pone.0047995

**Published:** 2012-10-31

**Authors:** Elizabeth Iorns, Katherine Drews-Elger, Toby M. Ward, Sonja Dean, Jennifer Clarke, Deborah Berry, Dorraya El Ashry, Marc Lippman

**Affiliations:** 1 Department of Medicine and Division of Hematology and Oncology, University of Miami Miller School of Medicine, Miami, Florida, United States of America; 2 Stanford Cancer Institute (SCI), Stanford University School of Medicine, Stanford, California, United States of America; 3 Science Exchange Inc., Palo Alto, California, United States of America; 4 Histopathology & Tissue Shared Resource, Lombardi Comprehensive Cancer Center, Georgetown University, Washington, D.C., United States of America; University of Kentucky College of Medicine, United States of America

## Abstract

Breast cancer is the most common cancer in women, and this prevalence has a major impact on health worldwide. Localized breast cancer has an excellent prognosis, with a 5-year relative survival rate of 85%. However, the survival rate drops to only 23% for women with distant metastases. To date, the study of breast cancer metastasis has been hampered by a lack of reliable metastatic models. Here we describe a novel in vivo model using human breast cancer xenografts in NOD *scid* gamma (NSG) mice; in this model human breast cancer cells reliably metastasize to distant organs from primary tumors grown within the mammary fat pad. This model enables the study of the entire metastatic process from the proper anatomical site, providing an important new approach to examine the mechanisms underlying breast cancer metastasis. We used this model to identify gene expression changes that occur at metastatic sites relative to the primary mammary fat pad tumor. By comparing multiple metastatic sites and independent cell lines, we have identified several gene expression changes that may be important for tumor growth at distant sites.

## Introduction

Metastatic disease is responsible for greater than 80% of all deaths from carcinoma [1]. Therefore a central goal of cancer research is to identify and characterize mechanisms that drive metastasis, to allow the development of new therapeutic agents to inhibit metastasis.

Metastasis is a complex process involving multiple steps by which tumor cells escape from the primary site and disseminate to form new lesions in other organs. To metastasize, tumor cells must degrade and cross the extracellular matrix, intravasate, travel through blood or lymphatic vessels, extravasate at the secondary site, and finally, establish secondary tumors [2]. To understand metastasis it is important that research models replicate the many steps involved in this complex process.

Previous studies into breast cancer metastasis have been limited by models that poorly replicate the entire metastatic process [3], [4]. An ideal model would reproduce the entire metastatic process, including tumor cell escape and dissemination from the orthotopic site (i.e., the mouse mammary fat pad), followed by colonization and outgrowth at distant sites. The most commonly used model of breast cancer metastasis relies on injecting tumor cells directly into the circulation via the tail vein or left ventricle of the heart [5], [6]. This model removes the requirement for cells to invade and intravasate, and therefore cannot be used to study the complete metastatic process. Thus, new models that replicate the metastatic process in its entirety are required to improve our understanding of how breast cancer spreads.

Here we describe a new system to study breast cancer metastasis that models the entire metastatic process. Using orthotopic injection of human breast cancer cells into the mammary fat pads of NOD *scid* gamma (NSG) mice [7], metastases in distant organs consistently develop without the need for resection of the primary tumor. Therefore, this model can be used to study the metastatic process in its entirety. We used this model to identify gene expression changes that occur in metastatic lesions compared to the primary tumor. These gene expression changes may regulate metastatic growth at distant sites and may represent potential therapeutic targets to inhibit metastatic disease.

## Results

### NSG mice are highly susceptible to metastasis formation

**Figure 1:**
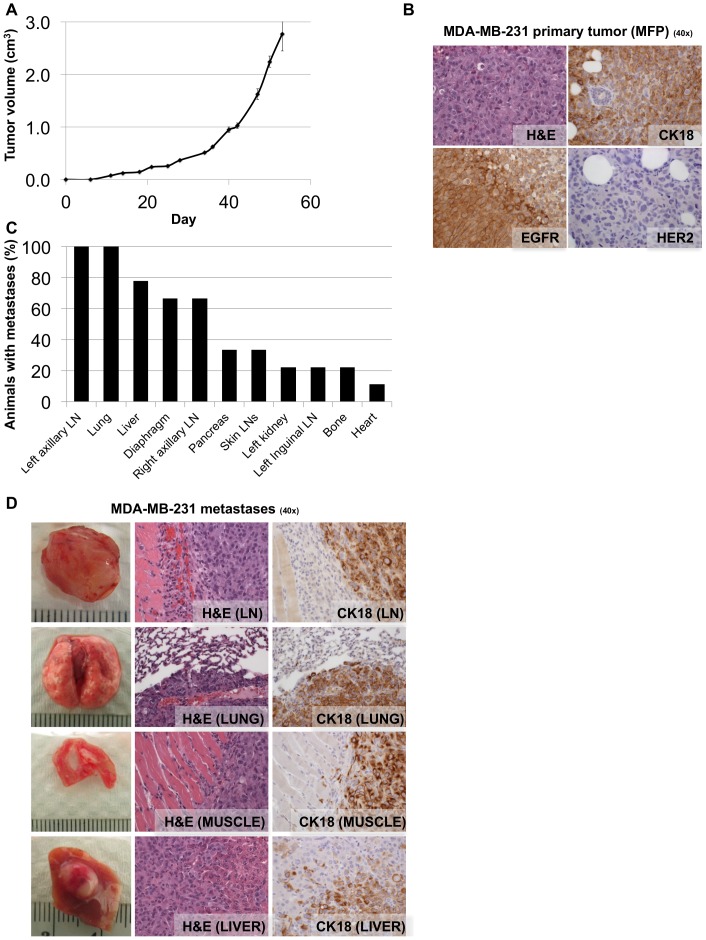
NSG mice consistently develop widespread macro-metastases when MDA-MB-231 cells are injected orthotopically into the mammary fat pad. **A.** Volumes of tumors in mammary fat pads of NSG mice injected with MDA-MB-231 cells. Each data point is the mean value (+/− s.e.m) of nine primary tumors. **B.** Micrographs of haematoxylin and eosin (H&E), CK18, EGFR and Her2 IHC staining of harvested MDA-MB-231 primary tumor tissue. MDA-MB-231 primary tumors are CK18 positive, EGFR positive, Her2 negative. **C.** Quantification of the percentage of mice bearing macro-metastasis in each organ observed at the time of necropsy (53 days post injection). Macro-metastases were frequently observed in the left axillary lymph node (100% of mice), lung (100% of mice), liver (78% of mice), diaphragm (67% of mice), and right axillary lymph node (67% of mice), as well as sporadically in other organs. LN = lymph node. **D.** Photographs of representative common MDA-MB-231 metastases are shown, along with micrographs of H&E and CK18 IHC staining of harvested tissue.

In order to develop a system that models the entire metastatic process, we injected aggressive basal MDA-MB-231 human breast carcinoma cells orthotopically into the mammary fat pads of nine severely immunocompromised NSG mice. Mice were monitored for development of primary xenograft tumors and sacrificed when tumors reached 10% of body weight. Following injection, the primary mammary fat pad tumors grew rapidly (Figure 1A), resulting in sacrifice of the mice at day 53 post-injection. Harvested primary mammary fat pad tumor tissue was immunohistochemically stained for CK18, EGFR and Her2 expression. MDA-MB-231 primary tumors were CK18 positive, EGFR positive, Her2 negative (Figure 1B), demonstrating that the xenograft tumors retained the MDA-MB-231 cell line expression pattern of key breast cancer markers. At necropsy mice were examined visually for macro-metastases. Macro-metastases were frequently and consistently observed in axillary lymph nodes (100% of mice), lungs (100% of mice), liver (78% of mice), and diaphragm (67% of mice), as well as sporadically in other organs (Figure 1C). Organs were harvested and the presence of metastases was confirmed by H&E and CK18 staining of tissue sections (Figure 1D).

**Figure 2:**
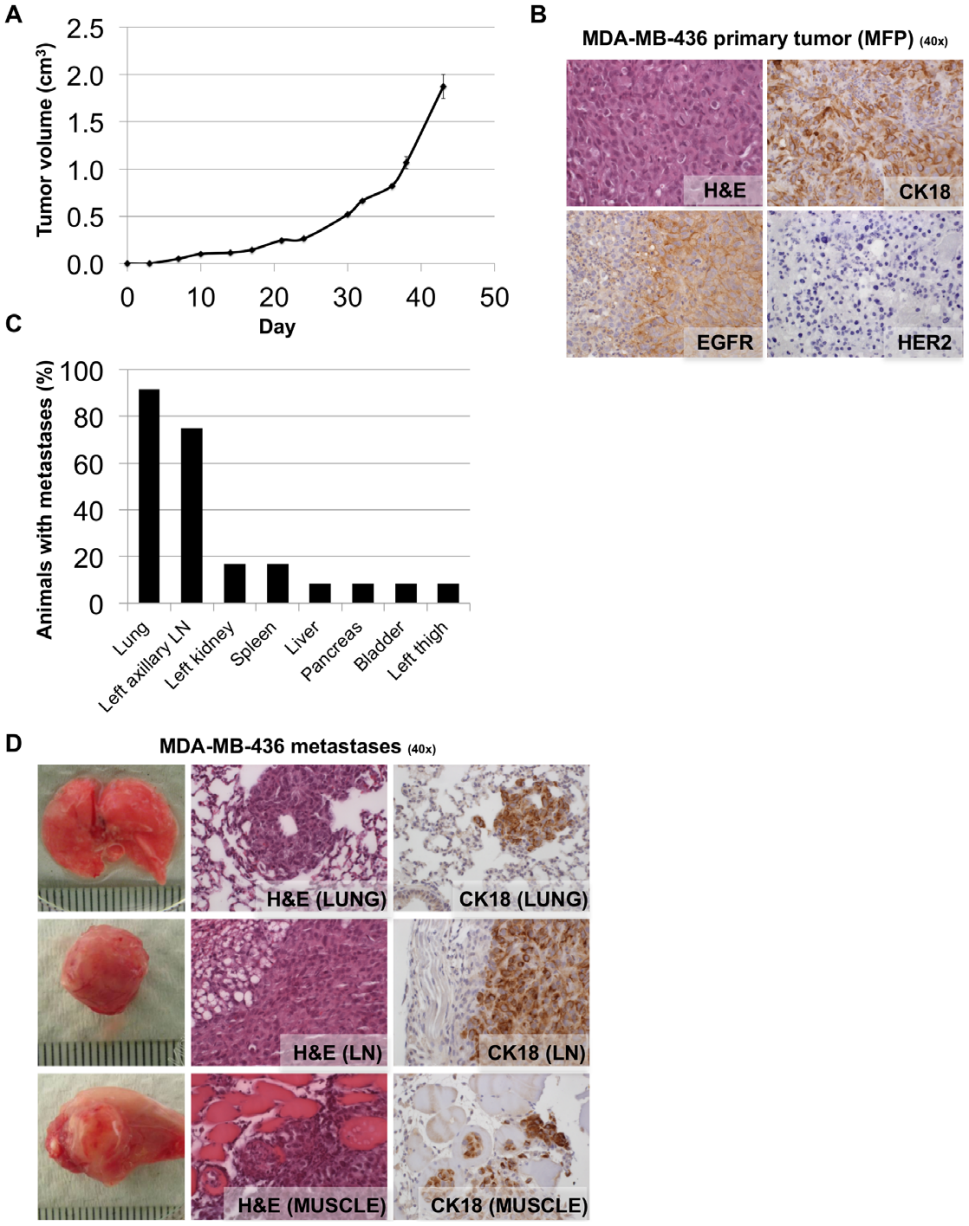
NSG mice consistently develop macro-metastases when MDA-MB-436 cells are injected orthotopically into the mammary fat pad. **A.** Volumes of tumors in mammary fat pads of NSG mice injected with MDA-MB-436 cells. Each data point is the mean value (+/− s.e.m) of 12 primary tumors. **B.** Micrographs of haematoxylin and eosin (H&E), CK18, EGFR and Her2 IHC staining of harvested MDA-MB-436 primary tumor tissue. MDA-MB-436 primary tumors are CK18 positive, EGFR positive, Her2 negative. **C.** Quantification of the percentage of mice bearing macro-metastasis in each organ observed at the time of necropsy (43 days post injection). Macro-metastases were frequently observed in the lung (92% of mice) and left axillary lymph node (75% of mice), as well as sporadically in other organs. LN = lymph node. **D.** Photographs of representative common MDA-MB-436 metastases are shown, along with micrographs of H&E and CK18 IHC staining of harvested tissue.

To determine if this high rate of metastasis was restricted to MDA-MB-231 cells, we injected twelve NSG mice with another aggressive basal breast carcinoma cell line, MDA-MB-436. Similarly to MDA-MB-231 cells, MDA-MB-436 cells rapidly formed primary tumors in the mammary glands of female NSG mice (Figure 2A). Similarly to MDA-MB-231, harvested primary mammary fat pad tumor tissue was immunohistochemically stained for CK18, EGFR and Her2 expression. MDA-MB-436 primary tumors were CK18 positive, EGFR positive, Her2 negative (Figure 2B), demonstrating that the NSG MDA-MB-436 xenograft tumors also retained the MDA-MB-436 cell line expression pattern of key breast cancer markers. Upon necropsy at day 43 macro-metastases were frequently observed in the lungs and axillary lymph nodes of tumor bearing mice (Figure 2C). Organs were harvested and the presence of metastases was confirmed by H&E and CK18 staining (Figure 2D).

**Figure 3:**
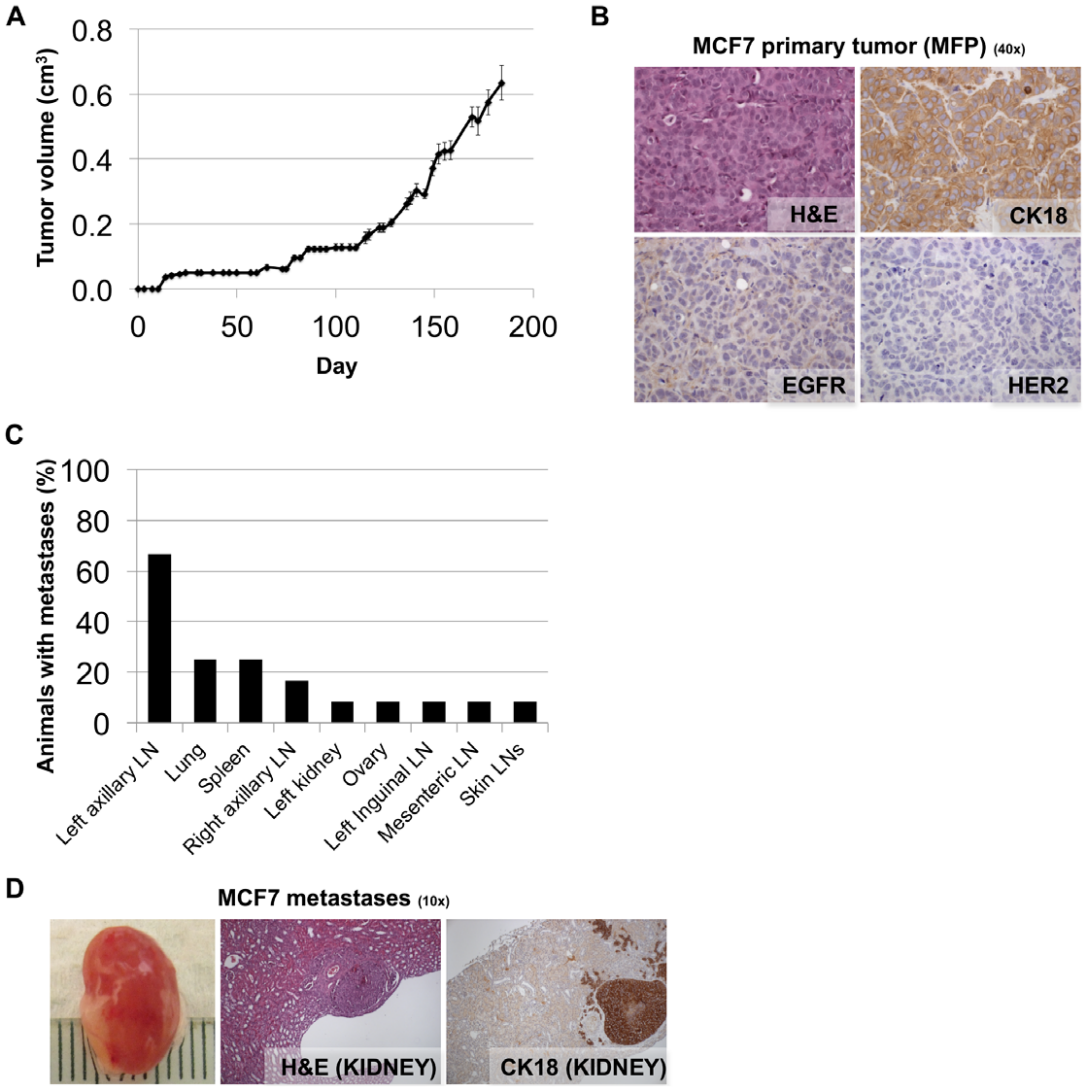
NSG mice occasionally develop macro-metastases when MCF7 cells are injected orthotopically into the mammary fat pad. **A.** Volumes of tumors in mammary fat pads of NSG mice injected with MCF7 cells. Each data point is the mean value (+/− s.e.m) of 12 primary tumors. **B.** Micrographs of haematoxylin and eosin (H&E), CK18, EGFR and Her2 IHC staining of harvested MCF7 primary tumor tissue. MCF7 primary tumors are CK18 positive, EGFR negative, Her2 negative. **C.** Quantification of the percentage of mice bearing macro-metastasis in each organ observed at the time of necropsy (184 days post injection). Macro-metastases were observed in the left axillary lymph node (67% of mice), lung (25% of mice), and spleen (25% of mice), as well as sporadically in other organs. LN = lymph node. **D.** A photograph of an MCF7 kidney metastasis is shown, along with micrographs of H&E and CK18 IHC staining of harvested tissue.

Having confirmed that aggressive basal breast carcinoma cell lines frequently metastasize in NSG mice, we examined whether the luminal MCF7 and BT474 breast carcinoma cell lines could metastasize in this model. Metastases are almost never observed with these two cell lines in other strains of mice [8]. MCF7 cells formed slow growing primary tumors in the mammary gland (Figure 3A) that were CK18 positive, EGFR negative, Her2 negative (Figure 3B). Interestingly MCF7 cells frequently metastasized to the axillary lymph node (67% of mice), lung (25% of mice), and spleen (25% of mice) (Figure 3C). We also observed sporadic metastases including a kidney lesion (Figure 3D) and a large ovarian metastasis, which are very unusual.

**Figure 4:**
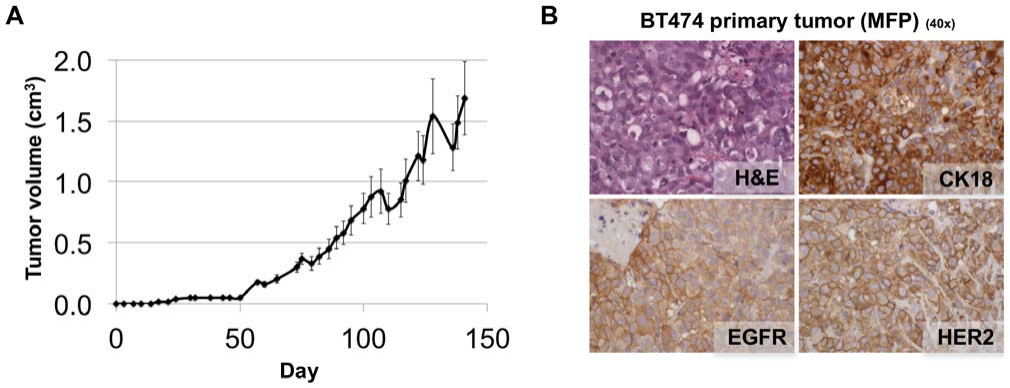
NSG mice occasionally develop macro-metastases when BT474 cells are injected orthotopically into the mammary fat pad. **A.** Volumes of tumors in mammary fat pads of NSG mice injected with BT474 cells. Each data point is the mean value (+/− s.e.m) of 12 primary tumors. **B.** Micrographs of haematoxylin and eosin (H&E), CK18, EGFR and Her2 IHC staining of harvested BT474 primary tumor tissue. BT474 primary tumors are CK18 positive, EGFR positive, Her2 positive.

BT474 cells formed primary mammary fat pad tumors (Figure 4A) that were CK18 positive, EGFR positive, Her2 positive (Figure 4B). BT474 cells were much less metastatic in this model, forming macro-metastases in only a few cases (axillary lymph node in 17% of mice, and spleen in 8% of mice). Importantly, these cells come from a solid invasive ductal carcinoma of the breast with no known metastatic disease [9]. Thus, the metastatic ability of the original tumor is recapitulated by our model.

We also confirmed that macro-metastasis formation was not restricted to established cell lines by using a primary breast cancer culture, dissociated tumor (DT)-16 [10]. Similarly to established cell lines, DT-16 primary breast cells also frequently metastasized to the liver (3/3) and lymph nodes (3/3) of NSG mice.

Having determined that MDA-MB-231 and MDA-MB-436 breast cancer cell lines frequently and reliably metastasize to distant organs in NSG mice, we used these models to examine in vivo gene expression alterations that occur in metastatic lesions relative to the primary mammary fat pad tumors.

**Figure 5:**
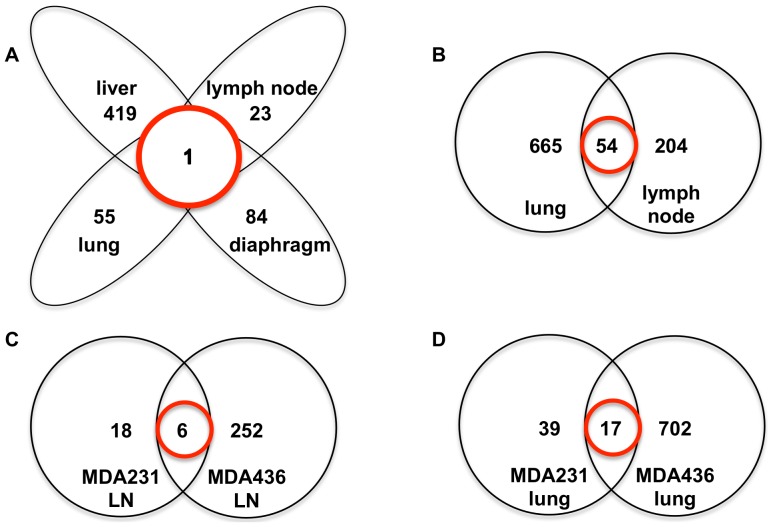
Transcriptional changes in MDA-MB-231 and MDA-MB-436 macro-metastases relative to primary tumors. **A.** Venn diagram of genes differentially expressed in MDA-MB-231 liver, lung, diaphragm and lymph node metastases compared to primary mammary fat pad tumor (n = 3 mice per group). One probe (Table S5) is differentially expressed in all metastases compared to the primary mammary fat pad tumor (indicated by the red circle). Cut offs used for gene list generation: p value with FDR<0.01. **B.** Venn diagram of genes differentially expressed in MDA-MB-436 lung and lymph node metastases compared to primary mammary fat pad tumor (n = 3 mice per group). 54 probes (Table S8) are differentially expressed in both lung and lymph node metastases compared to the primary mammary fat pad tumor (indicated by the red circle). Cut offs used for gene list generation: p value with FDR<0.01. **C.** Venn diagram of genes differentially expressed in MDA-MB-231 and MDA-MB-436 lymph node metastases compared to primary mammary fat pad tumors. Six probes (Table S9) are differentially expressed in both lymph node metastases compared to their respective primary mammary fat pad tumors. **D.** Venn diagram of genes differentially expressed in MDA-MB-231 and MDA-MB-436 lung metastases compared to primary mammary fat pad tumors. 17 probes (Table S10) are differentially expressed in both lung metastases compared to their respective primary mammary fat pad tumors.

We compared the gene expression profiles of MDA-MB-231 metastatic lesions from several organs (liver, lung, diaphragm, and lymph nodes) to the gene expression profile of the primary xenograft tumor growing in the mammary fat pad of NSG mice. 420 genes were differentially expressed in liver metastases, 56 in lung metastases, 85 in diaphragm metastases, and 24 in lymph node metastases, compared to the primary mammary fat pad tumor (Tables S1, S2, S3, and S4). These differentially expressed genes were then compared and a single gene, LOC100129599, was identified as consistently differentially expressed at all metastatic sites relative to the primary tumor (Figure 5A and Table S5).

Similarly, the gene expression profile of MDA-MB-436 metastatic lesions growing in lung and lymph nodes were also compared to the gene expression profile of the primary xenograft mammary fat pad tumor. 719 genes were differentially expressed in lung metastases, and 258 in lymph node metastases, compared to the primary mammary fat pad tumor (Tables S6 and S7). 54 of the gene expression changes were common to both the lung and lymph node metastases (Figure 5B and Table S8). LOC100129599 was differentially expressed in the MDA-MB-436 lymph node metastases relative to the primary mammary fat pad tumor, but not the lung metastases.

The gene expression changes that occurred in lymph node metastases relative to primary mammary fat tumor in MDA-MB-231 xenografts were then compared to those that occurred in MDA-MB-436 xenografts to detect any commonalities (Figure 5C and Table S9). Six genes, RPL13A, KLF6, LOC100129599, GBE1, PDGFA, and UHRF1 were altered in lymph node metastases of both cell lines relative the primary tumor, suggesting they may represent changes important for metastatic growth in lymph nodes.

Similarly, the gene expression changes that occurred in lung metastases relative to primary mammary fat tumors in MDA-MB-231 xenografts were compared to those that occurred in MDA-MB-436 xenografts to detect any commonalities (Figure 5D and Table S10). 17 probes (16 genes) were altered in lung metastases of both cell lines relative to the primary tumor suggesting they may represent changes important for metastatic growth in the lungs.

## Discussion

Human breast cancer cell lines derived from tumors with metastatic capability in the original patient rarely metastasize in commonly used immunocompromised mouse models, including nude and NOD SCID mice. This lack of reliable metastasis in vivo has hampered thorough investigation of the metastatic process. Our use of NSG mice represents a significant advance, due to the ability of cancer cells including MDA-MB-231, MDA-MB-436, MCF7, and dissociated primary breast cancer cells (DT-16) to reliably form distant metastatic lesions after primary tumor formation in the orthotopic mammary fat pad.

Previously, the occurrence of metastasis in mouse models using human breast cancer cells was sporadic and occurred only at low rates, even when cells were injected into the tail vein or administered via the intracardiac route. In BALB/c nude mice, MDA-MB-231 cells injected into the mammary fat pad yielded no lung metastases in five mice tested [5]. Only after being “trained” through serial tail-vein injections and harvesting of lung lesions were the cells able to metastasize from the mammary fat pad, and then in less than 50% of mice [5].

The more severely immunocompromised NOD SCID mice have been used to try to obtain a more robust breast cancer metastasis model. However, metastasis formation by MDA-MB-231 cells in NOD SCID mice can only generally be accomplished via tail-vein injection, and not by injection into the orthotopic mammary fat pad [11]. In addition, the formation of metastases when MDA-MB-231 cells were injected into the tail-vein was limited to the lungs.

When compared to these rates of metastasis, MDA-MB-231 cells in our NSG model form macro-metastases much more frequently and consistently (axillary lymph nodes 100% of mice, lungs 100% of mice, liver 78% of mice, and diaphragm 67% of mice), providing a robust model for the study of human breast cancer metastasis in mice.

The mechanism(s) limiting cancer cell metastasis in other mouse models have been frequently debated, and has been hypothesized to be due to a wide variety of reasons, ranging from the smaller size of the murine vasculature, to human-mouse signaling incompatibilities, to selection of non-metastatic subpopulations of breast cancer cells during culture. Here we show that human breast cancer cells frequently metastasize in severely immunocompromised NSG murine hosts, indicating that the immune environment (at least partly) determines the metastatic ability of the human breast cancer cells. NSG mice lack functional B, T and Natural Killer (NK) cell populations; in contrast, the more commonly used strains of mice such as nudes and NOD SCID mice still retain NK (and B cells in the case of nude mice). This suggests that NSG mice may be inherently susceptible to metastasis due to the lack of NK cells, demonstrating the importance of these cells in regulating the metastatic process.

We used the NSG model to identify gene expression changes that occur in metastatic lesions relative to primary tumors. When compared, a single gene, LOC100129599, was the most consistently differentially expressed gene at the majority of metastatic sites relative to primary tumors, in both MDA-MB-231 and MDA-MB-436 metastatic lesions. LOC100129599, also known as ribosomal protein S29 pseudogene 14 (RPS29P14), is a pseudogene, and noncoding RNAs transcribed from pseudogenes have been implicated in tumor progression previously [12]. Thus, it is possible that altered expression of LOC100129599 may regulate metastasis and this will be investigated in future experiments.

After obtaining differential expression data from lymph node and lung metastases relative to primary tumors from two independent cell lines, we compared these two datasets to identify commonalities. Six genes, RPL13A, KLF6, LOC100129599, GBE1, PDGFA, and UHRF1 were altered in lymph node metastases of both cell lines relative to the primary tumors, suggesting they may represent changes important for metastatic growth in lymph nodes.

Literature searches were performed to determine if any of these genes have previously been implicated in breast cancer lymph node metastasis. Interestingly, overexpression of PDGFR-α expression in breast cancer has previously been associated with lymph node metastasis (*P* = 0.0079) in a cohort of 181 invasive ductal breast carcinomas patients [13] suggesting that PDGFA may be clinically relevant for breast cancer lymph node metastasis. In addition, expression of PGDF has been shown to be associated with poor clinical outcome in two further studies of breast cancer patients [14], [15]. Evidence supporting a functional role for PDGF in mediating lymph node metastasis is provided by a study demonstrating that members of the PDGF family act as lymphangiogenic factors. In this study, expression of PDGF-BB induced tumor lymphangiogenesis, leading to enhanced metastasis in lymph nodes [16].

UHRF1 was also identified from our literature search as a clinically relevant marker of lymph node metastatic capacity, in this case in colorectal carcinoma samples. UHRF1 protein expression was determined in 144 clinical colorectal carcinoma samples.

UHRF1 protein expression levels correlated with the presence of lymph nodes (*P* = 0.005), and distal metastasis (*P* = 0.030) [17]. In addition, UHRF1 was shown to induce angiogenesis of MDA-MB-231 xenografts (assessed by microvessel density) and increase proliferation, invasion and migration in in vitro assays [18] suggesting a functional role for UHRF1 in regulating metastasis.

The published demonstration of clinical and functional roles for two of the genes identified from our microarray analysis of lymph node metastases suggests that the changes we have identified may represent alterations important for metastatic growth in lymph nodes, and bolsters the relevance of the NSG metastasis model.

Next, the gene expression changes that occurred in lung metastases relative to primary mammary fat tumor in MDA-MB-231 xenografts were compared to those that occurred in MDA-MB-436 xenografts to determine any commonalities. 17 probes (detecting 16 genes) were altered in lung metastases of both cell lines relative to primary tumors. Of these 16 genes, RPL14L (downregulated in lung metastases in both cell lines) has previously been identified as a candidate breast cancer metastasis regulating gene [19], and loss of TGFBR2 (downregulated in lung metastases in both cell lines) has previously been shown to promote metastasis in mouse models [20]. This suggests that the changes we have identified may represent alterations important for metastatic growth in the lung, similarly to the changes we identified for metastatic growth in lymph nodes.

Previously a lung tropic metastasis signature was identified by training MDA-MB-231 cells to form lung metastases in nude mice [5]. We first compared this to our 16 gene lung metastasis signature (distilled from MDA-MB-231 and MDA-MB-436 data) to determine if any genes were shared in both signatures. Interestingly, there were no common genes identified. We next compared the lung-tropic signature to our 56 gene signature generated solely from MDA-MB-231 lung metastases. One gene, collagen, type VI, alpha 1 (COL6A1), was up-regulated in both MDA-MB-231 signatures. Previous studies have shown that COL6A1 promotes metastasis [21] so it is likely that COL6A1 is particularly important for lung metastasis in MDA-MB-231 cells. It is possible that gene changes facilitating early events in the metastatic cascade (escape from the primary site, invasion, and intravasation) that are captured in our orthotopic model are not represented in the “trained” MDA-MB-231 lung signature, which may explain why there is not more overlap between the two signatures.

In summary, the NSG mouse is an important new model for studying breast cancer metastasis, providing an efficient system to study the entire metastatic process from the mammary gland using human cells. This model should facilitate better understanding of the fundamental mechanisms underlying breast cancer metastasis, which may ultimately lead to important therapeutic advances.

## Experimental Procedures

### Ethics statement

The animal experiments described in this study were approved by the Institutional Animal Care and Use Committee (IACUC) at the University of Miami (protocol 11–227). All animals were maintained in accordance with IACUC guidelines.

### Cell lines

BT474, MCF7, MDA-MB-231 and MDA-MB-436 human breast carcinoma cell lines were obtained from the American Type Culture Collection (Manassas, VA) and maintained according to the supplier's instructions. Authentication of the cell lines was performed by sequencing of the hypervariable regions of the mitochondrial DNA. DT-16 cells were obtained and cultured as described previously [10]. Cells were harvested at the exponential phase of growth for injection into the mammary fat pads of mice.

### Animals

NOD *scid* gamma (NSG) mice were purchased from Jackson Laboratory. Eight week old female mice were injected unilaterally with 2.5×10^6^ cells in 200 µL of 50∶50 Matrigel/Collagen I into the fourth abdominal fat pad by subcutaneous injection at the base of the nipple. Tumor growth was monitored externally using vernier calipers for up to 30 weeks and animals sacrificed when tumors reached 10% of body weight. Necropsies were performed to identify macro-metastases. Primary tumors and organs were harvested, paraffin embedded, sectioned and stained with hematoxylin and eosin or antibodies against cytokeratin 18 (CK18), epidermal growth factor receptor (EGFR), and Her2. Pathology processing and staining of harvested mouse tissues was performed at the Lombardi Comprehensive Cancer via Science Exchange, Inc. Slides were analyzed by a pathologist to confirm the presence of metastases. RNA was isolated from three independent metastases and primary xenograft tumors for analysis. Note: No estrogen pellets were required for growth of estrogen receptor alpha positive cell lines, MCF7 and BT474.

### RNA isolation

RNA was extracted from xenograft primary and metastatic lesions using Trizol Reagent (Invitrogen) according to the manufacturer's instructions. Concentration and yield of RNA samples were determined using a NanoDrop ND-1000 Spectrophotometer (NanoDrop Technologies). RNA integrity was determined by analysis on an Agilent 2100 Bioanalyzer (Agilent Technologies) following the manufacturer's recommendations. Only samples with a RIN score >7.0 were used for microarray analysis.

### Microarray Analysis

The human Illumina gene expression image files obtained from the Illumina iScan scanner were uploaded to GenomeStudio (version 2011.1) using the Gene Expression module (v1.9.0). Data were normalized using the quantile method and the background was subtracted. Differentially expressed probes were identified using the Illumina Custom Error Model with Benjamini and Hochberg False Discovery Rate. A p-value was associated with every probe (detection p-value) and probes were discarded if this detection p value was more than 0.05 for all samples. Metastatic gene expression alterations were determined by identifying genes differentially expressed between mammary fat pad tumor xenografts and metastases. The differentially expressed genes from comparisons were used to create Venn diagrams. Microarray experiments and analyses of datasets were performed at Wayne State via Science Exchange, Inc.

## Supporting Information

Table S1 **Transcripts differentially expressed (p<0.01) in MDA-MB-231 liver metastases compared to primary mammary fat pad tumor xenografts in NSG mice (total 420).**
(XLSX)

Table S2 **Transcripts differentially expressed (p<0.01) in MDA-MB-231 lung metastases compared to primary mammary fat pad tumor xenografts in NSG mice (total 56).**
(XLSX)

Table S3 **Transcripts differentially expressed (p<0.01) in MDA-MB-231 diaphragm metastases compared to primary mammary fat pad tumor xenografts in NSG mice (total 85).**
(XLSX)

Table S4 **Transcripts differentially expressed (p<0.01) in MDA-MB-231 lymph node metastases compared to primary mammary fat pad tumor xenografts in NSG mice (total 24).**
(XLSX)

Table S5 **Venn diagram of genes differentially expressed in MDA-MB-231 liver, lung, diaphragm and lymph node metastases compared to primary mammary fat pad tumor xenografts in NSG mice.** 1 probe is differentially expressed in all metastases compared to the primary mammary fat pad tumor.
(XLSX)

Table S6 **Transcripts differentially expressed (p<0.01) in MDA-MB-436 lung metastases compared to primary mammary fat pad tumor xenografts in NSG mice (total 719).**
(XLSX)

Table S7 **Transcripts differentially expressed (p<0.01) in MDA-MB-436 lymph node metastases compared to primary mammary fat pad tumor xenografts in NSG mice (total 258).**
(XLSX)

Table S8 **Venn diagram of genes differentially expressed in MDA-MB-436 lung and lymph node metastases compared to primary mammary fat pad tumor xenografts in NSG mice.** 54 probes are differentially expressed in both lung and lymph node metastases compared to the primary mammary fat pad tumor.
(XLSX)

Table S9 **Venn diagram of genes differentially expressed in MDA-MB-231 and MDA-MB-436 lymph node metastases compared to primary mammary fat pad tumors.** 6 probes are differentially expressed in both lymph node metastases compared to their respective primary mammary fat pad tumors.
(XLSX)

Table S10 **Venn diagram of genes differentially expressed in MDA-MB-231 and MDA-MB-436 lung metastases compared to primary mammary fat pad tumors.** 17 probes are differentially expressed in both lung metastases compared to their respective primary mammary fat pad tumors.
(XLSX)

## References

[1] Jemal A, Siegel R, Xu J, Ward E (2010) Cancer statistics, 2010. CA Cancer J Clin 60: 277–300. 20610543 10.3322/caac.20073

[2] Nguyen DX, Bos PD, Massagué J (2009) Metastasis: from dissemination to organ-specific colonization. Nat Rev Cancer 9: 274–84. 19308067 10.1038/nrc2622

[3] Price JE (1996) Metastasis from human breast cancer cell lines. Breast Cancer Res Treat 39: 93–102. 8738609 10.1007/BF01806081

[4] Francia G, Cruz-Munoz W, Man S, Xu P, Kerbel RS (2011) Mouse models of advanced spontaneous metastasis for experimental therapeutics. Nat Rev Cancer 11: 135–41. 21258397 10.1038/nrc3001 PMC4540342

[5] Minn AJ, Gupta GP, Siegel PM, Bos PD, Shu W, et al (2005) Genes that mediate breast cancer metastasis to lung. Nature 436: 518–24. 16049480 10.1038/nature03799 PMC1283098

[6] Kang Y, Siegel PM, Shu W, Drobnjak M, Kakonen SM, et al (2003) A multigenic program mediating breast cancer metastasis to bone. Cancer Cell 3: 537–49. 12842083 10.1016/s1535-6108(03)00132-6

[7] Shultz LD, Lyons BL, Burzenski LM, Gott B, Chen X, et al (2005) Human lymphoid and myeloid cell development in NOD/LtSz-scid IL2R gamma null mice engrafted with mobilized human hemopoietic stem cells. J Immunol 174: 6477–89. 15879151 10.4049/jimmunol.174.10.6477

[8] Lee TH, Seng S, Sekine M, Hinton C, Fu Y, et al (2007) Vascular endothelial growth factor mediates intracrine survival in human breast carcinoma cells through internally expressed VEGFR1/FLT1. PLoS Med 4: e186. 17550303 10.1371/journal.pmed.0040186 PMC1885450

[9] Lasfargues EY, Coutinho WG, Redfield ES (1978) Isolation of two human tumor epithelial cell lines from solid breast carcinomas. J Natl Cancer Inst 61: 967–78. 212572

[10] Bayliss J, Hilger A, Vishnu P, Diehl K, El-Ashry D (2007) Reversal of the estrogen receptor negative phenotype in breast cancer and restoration of antiestrogen response. Clin Cancer Res 13: 7029–36. 18056179 10.1158/1078-0432.CCR-07-0587

[11] Kuperwasser C, Dessain S, Bierbaum BE, Garnet D, Sperandio K, et al (2005) A mouse model of human breast cancer metastasis to human bone. Cancer Res 65: 6130–8. 16024614 10.1158/0008-5472.CAN-04-1408

[12] Prensner JR, Chinnaiyan AM (2011) The emergence of lncRNAs in cancer biology. Cancer Discov 1: 391–407. 22096659 10.1158/2159-8290.CD-11-0209 PMC3215093

[13] Carvalho I, Milanezi F, Martins A, Reis RM, Schmitt F (2005) Overexpression of platelet-derived growth factor receptor alpha in breast cancer is associated with tumour progression. Breast Cancer Res 7: R788–95. 16168125 10.1186/bcr1304 PMC1242156

[14] Seymour L, Dajee D, Bezwoda WR (1993) Tissue platelet derived-growth factor (PDGF) predicts for shortened survival and treatment failure in advanced breast cancer. Breast Cancer Res Treat 26: 247–52. 8251649 10.1007/BF00665802

[15] Seymour L, Bezwoda WR (1994) Positive immunostaining for platelet derived growth factor (PDGF) is an adverse prognostic factor in patients with advanced breast cancer. Breast Cancer Res Treat 32: 229–33. 7865852 10.1007/BF00665774

[16] Cao R, Björndahl MA, Religa P, Clasper S, Garvin S, et al (2004) PDGF-BB induces intratumoral lymphangiogenesis and promotes lymphatic metastasis. Cancer Cell 6: 333–45. 15488757 10.1016/j.ccr.2004.08.034

[17] Wang F, Yang YZ, Shi CZ, Zhang P, Moyer MP, et al (2012) UHRF1 Promotes Cell Growth and Metastasis Through Repression of p16(ink4a) in Colorectal Cancer. Ann Surg Oncol 19: 2753–62. 22219067 10.1245/s10434-011-2194-1

[18] Li XL, Xu JH, Nie JH, Fan SJ (2012) Exogenous expression of UHRF1 promotes proliferation and metastasis of breast cancer cells. Oncol Rep 28: 375–83. 22552622 10.3892/or.2012.1792

[19] Driouch K, Lidereau R (2000) Molecular analyses of human breast cancer metastasis: genetic markers of progression. Breast Cancer Res 2: P7.01.

[20] Fang WB, Jokar I, Chytil A, Moses HL, Abel T, et al (2011) Loss of one Tgfbr2 allele in fibroblasts promotes metastasis in MMTV: polyoma middle T transgenic and transplant mouse models of mammary tumor progression. Clin Exp Metastasis 28: 351–66. 21374085 10.1007/s10585-011-9373-0 PMC3373018

[21] Chiu KH, Chang YH, Wu YS, Lee SH, Liao PC (2011) Quantitative secretome analysis reveals that COL6A1 is a metastasis-associated protein using stacking gel-aided purification combined with iTRAQ labeling. J Proteome Res 10: 1110–25. 21186846 10.1021/pr1008724

